# Associations between emotional intelligence, empathy and personality in Japanese medical students

**DOI:** 10.1186/s12909-018-1165-7

**Published:** 2018-03-27

**Authors:** Keiko Abe, Masayuki Niwa, Kazuhiko Fujisaki, Yasuyuki Suzuki

**Affiliations:** 1 Medical Educational Development Center, Gifu University, Gifu, Japan; 2 College of Nursing, Aichi Medical University, Nagakute, Japan

**Keywords:** Emotional intelligence, Empathy, Personality, Medical student

## Abstract

**Background:**

It is known that empathic communication is important for physicians to achieve higher patient satisfaction and health outcomes. Emotional intelligence (EI), empathy and personality in medical students predict students’ individual disposition and their emotional and empathic perceptions. This study aimed to investigate: 1) The association between empathy, EI and personality, and 2) Gender differences in the association between empathy, EI and personality.

**Method:**

Participants were 357 1st year medical students from 2008 to 2011 at one medical school in Japan. Students completed self-report questionnaires comprising three validated instruments measuring EI: Trait Emotional Intelligence Questionnaire-Short Form (TEIQue-SF), empathy: Jefferson Scale of Physician Empathy- student version (JSPE) and personality: NEO-Five-Factor Inventory (NEO-FFI), which explores 5 dimensions of personality Neuroticism (N), Extraversion (E), Openness to experience (O), Agreeableness (A), and Conscientiousness (C).

**Results:**

Pearson Correlations showed weak association between TEIQue-SF and JSPE. TEIQue-SF and NEO-FFI showed positive correlation for E and C, and strong negative correlation for N and weak positive correlation for A and O. Weak positive correlation between JSPE and the NEO-FFI were observed for E and A. Although effect sizes were small, N, A and empathy were significantly higher in females (unpaired t-test). However, hierarchical multiple-regression analysis when controlling for gender and personality showed no association between EI, empathy and gender. A, TEIQue-SF and N were found to make small contributions in respect of predictions for JSPE. Personality contributed significantly to the prediction of TEIQue-SF. N had the largest independent negative contribution (β = − 0,38).

**Conclusion:**

In our study population of 1st year medical students, females had significantly higher N, A and empathy scores than males. Medical students’ N score was strongly negatively associated with EI. Empathy was weakly associated with EI and A. However, when controlling gender and personality in regression analysis, gender did not affect EI and empathy, rather personality is the most important factor. Our findings indicate that N is a major factor that negatively affects EI. It is important to mitigate N using thoughtful training, taking into account students’ personalities, to reduce N. In future studies, we will assess how communication trainings for students might enhance EI.

## Background

Compassion and empathy during patient-physician communication are major components in successful treatment of health problems [1]. Thus, empathic physicians are more likely to produce improved patient-physician relationship, patient trust, satisfaction, compliance and to achieve better outcomes in clinical practices [2–4]. In addition, these patients’ positive outcomes prompt physicians to improve their clinical decisions, leading to greater job satisfaction and well-being, and better leadership skills within their healthcare team [5–7]. As well as biomedical knowledge and clinical skills, physician performance is strongly influenced by their empathetic and emotional abilities, which in turn may be shaped and influenced by personality.

In the USA, the Association of American Medical Colleges (AAMC) states in their learning objectives for medical school education that “physicians must be compassionate and empathetic in caring for patients” [8]. Additionally, for residents, the Accreditation Council for Graduate Medical Education (ACGME) has defined Emotional Intelligence (EI) competency as assessment methods of residents’ interpersonal and communication skills, professional behaviors and patient care which were 3 of 6 core competences for residents [9]. EI competency definitions include 12 abilities: Emotional self-awareness, Emotional self-control, Adaptability, Achievement orientation, Positive outlook, Empathy, Organizational awareness, Coach and mentor, Inspirational leadership, Influence, Conflict management, and Teamwork. ACGME concluded that competences of EI and empathy are essential components for 21st century physicians [10].

EI is a measure of emotional awareness and ability to respond to emotion in oneself and others. EI has two major concept models, namely *ability EI model* and *trait and mixed EI models*. *Ability EI* proposed by Mayer and Salovey is the ability to perceive and integrate emotion, as well as to understand and regulate emotion to promote personal growth [11]. It is measured by answering performance-based questions. And trait EI as described by Petrides and Furnam [12, 13] and mixed EI as described by Golemann [14] can be viewed as the set of interrelated competencies skills, abilities, personal qualities and personality trait. Trait EI measures one’s self-perception of emotional self-efficacy. Though the measurement of the construct is different, sampling domains overlap. Both models view EI as a multidimensional construct with cognitive and affective elements, including abilities to recognize, deal with and apply emotional information to everyday decision-making and behavior [15].

The measurement of Trait EI called TEIQue (Trail Emotional Intelligence Questionnaire) can provide vital and consistent cross-situational information about an individual’s personality and behavior, as a comprehensive measure of emotion-related self-perceptions [12]. Research has shown that higher EI positively contributes to the patient-physician relationship, increased empathy, teamwork and communication skills, stress management, organization commitment, leadership and higher academic performance [16–19].

EI and empathy have been found to be associated. Empathy is viewed as an ability to understand and share the feelings of another. It is a multi-dimensional construct which includes both cognitive and affective elements. Hojat defined empathy in the context of health care as: “predominantly a cognitive as opposed to affective of emotional attribute, that involves understanding of the patient’s pain, experiences, concerns and perspectives combined with a capacity to communicate this understanding and an intention to help” [20]. Thus, physicians’ empathic attitude is important in patients’ care to develop trustful patient-physician relationship and reduce their concerns.

In addition to EI and empathy, personality is another factor influencing the patient-physician relationship and patient care. Recently, both EI and empathy have been found to be related to personality in medical students and their ability as successful physicians. Costa et al. provided the Five-factor model for describing personality with 5 dimensions, which is called the Big Five: Neuroticism (N; anxiety, fearfulness and insecurity in relationships), Extraversion (E; sociability, positive affect and energetic behavior), Openness to experience (O; having active imagination and esthetic sensitivity), Agreeableness (A; altruistic, affective, and collaborative behaviors), and Conscientiousness (C; having self-discipline, persistence, and striving for achievement) [21]. O and A have been found to be positively correlated with empathy [22] and both A and E to social functioning [23], also E, O, A and C have been found to be highly positively correlated with EI, whereas, high N, a vulnerability factor for stress, negatively correlated with EI [24–26].

While there is clear evidence for an association between EI/empathy and improved patient care, there is also evidence that physicians with a high degree of empathy are at great risk of stress from emotional fatigue [27]. Because patient-physician relationships include an aspect of emotional labor, highly empathetic physicians may be at risk of ‘burn out’, and therefore they need to recognize this and learn emotional self-control. EI includes the ability not only to perceive and understand one’s own and other’s emotions, but it also includes the ability of how to manage emotions [12, 13].

Both EI and empathy have been found to be influenced by culture and by gender [13, 20]. These differences have been ascribed to communication patterns, including nonverbal communication, which may decrease empathic communication, as well as selection of individuals for medical schools, and to expectations of patients [24]. Females have generally been shown to be more empathetic [24, 25, 27–30]. Asian medical students frequently report lower empathy scores than Western medical students [19, 24, 28–30].

Because empathic behavior can be enhanced, medical students need to learn emotional skills to improve their perception, expression and control of their emotions in order to provide better patient care and improve teamwork with their colleagues in their future practices, as well as for their own well-being. Previous studies reported that communication training improved medical students’ EI and empathy [31–36]. Studies suggest that both EI and empathy can be enhanced through education. In addition, studies suggested that the personality of students should be taken into account in designing programs to enhance EI and empathy in undergraduate medical education [30, 32, 37]. For this reason, understanding the associations between empathy, EI and personality in this particular population may benefit those involved in medical education.

Thus, this study aims to investigate associations between EI, empathy, and personality in early period of Japanese first year medical students. This study posed the following 2 research questions:

1. What are the associations between EI, empathy and personality (5 dimensions)?
2. Do males and females differ in respect of the associations between EI, empathy and personality?

## Methods

### Participants

Each year between 2008 and 2011 first year medical students entering one Japanese medical school were invited to participate in a questionnaire-based survey. At total of 415 medical students voluntarily completed questionnaires comprising Japanese versions of three validated instruments.

### Procedure

We conducted the survey on the first day of the 2-day freshman medical school orientation camp, at a hotel conference room in the first week of their enrolment. After explaining the study objectives, the researchers distributed the three questionnaires. Then, those students who agreed to participate this study completed these three questionnaires. There was no penalty for any students who declined to participate in this study. Ethical approval was granted by Gifu University Graduate School of Medicine Ethics Committee. Anonymity and confidentiality for students were guaranteed.

### Instruments

We used three validated instruments (Table 1). The first measure was the Trait Emotional Intelligence Questionnaire-Short Form (TEIQue-SF) [13]. TEIQue-SF, which is available for academic and clinical research and has been translated into more than 20 languages, assesses how students perceive their ability to deal with their emotions to communicate with others. TEIQue-SF has 30 items with 7-point Likert scales (1 = strongly disagree, 7 = strongly agree) [13]. The second measure was the Jefferson Scale of Physician Empathy-student version (JSPE) [20], which is a reliable and extensively used tool (translated into 21 languages) to assesses students’ empathy, has 20 items; we reduced the 7-point Likert scale to a 5-point one (1 = strongly disagree, 5 = strongly agree), to save test completion time. Japanese versions of these instruments were validated by Abe et al. [38, 39]. The third measure was the NEO-Five Factor Inventory (NEO-FFI or the Big Five), which assesses features of 5 personality traits, has 60 items with 5-point Likert scales (1 = strongly disagree, 5 = strongly agree) [21], and translated into Japanese [40].

**Table 1:** Three kinds of measurement used in the study

Instrument	Abbreviation	No of items	Scale (Max scores)	Typical question
1) Trait Emotional Intelligence Questionnaire short Form	TEIQue-SF	30	1-7 (120)	I usually find it difficult to regulate my emotions.
2) Jefferson Scale of Physician Empathy-student version	JSPE	20	1-5 (100)	Patients feel better when their physicians understand their feelings.
3) BIG 5 Personality Inventory	NEO-FFI	60	0-4 (240)	
Neuroticism	N	12	0-4 (48)	I often feel tense and nervous.
Extraversion	E	12	0-4 (48)	I enjoy talking with friends.
Openness to experience	O	12	0-4 (48)	I do not feel anything when I read poems.
Agreeableness	A	12	0-4 (48)	I think I am a philanthropist.
Conscientiousness	C	12	0-4 (48)	I often find myself looking for things.

### Data analysis

Cronbach alpha was used to evaluate reliability of the questionnaires. The association between EI, empathy and each of 5 personality scores was assessed using Pearson’s Correlation Coefficient. Unpaired t-test was used to determine if there was a gender difference. The effect size was considered using Cohen’s d. Descriptive analysis of variables was circulated prior to the regression. Hierarchical multiple regression analysis was used to evaluate factors that influence EI and empathy scores (dependent variables) where control variables were gender and personality in the first block with independent variable of EI being empathy, and of empathy with EI being the independent variable in the second block. Statistical analyses were performed using SPSS, version 20.0 japan.

## Results

### Descriptive analysis

Participants’ response rates are shown in Table 2. Descriptive analysis, covering mean, variance, skewness and kurtosis for each instrument indicated no significant violation of normality in the distribution of each (Table 3).

**Table 2:** Participants and response rate in this study

	2008	2009	2010	2011	Total
Possible participants	96	102	109	108	415
Respondents (Response rate)	73 (76%)	85 (83%)	101 (93%)	98 (91%)	357 (86%)
Male	51 (70%)	63 (74%)	75 (74%)	76 (78%)	265 (74%)
Female	22 (30%)	22 (26%)	26 (26%)	22 (22%)	92 (26%)

**Table 3:** Descriptive analysis of mean, variance, skewness and kurtosis on TEIQue-SF, JSPE, age and the NEO-FFI

*n* = 357	Mean	SD	Variance	Skewness	Kurtosis
TEIQue-SF	132.81	20.83	433.79	−0.21	0.17
JSPE	83.89	8.95	80.15	−0.99	3.34
Age	20.42	4.89	23.91	2.77	8.20
N	25.77	7.97	63.54	0.04	−0.25
E	27.35	7.32	53.64	−0.22	−0.19
O	31.22	5.87	34.51	−0.17	0.43
A	32.03	5.41	29.31	−0.06	− 0.10
C	29.27	6.65	44.21	−0.32	−0.18

*TEIQue-SF* Trait Emotional Intelligence Questionnaire-Short Form, *JSPE* Jefferson Scale of Physician Empathy-student version, *N* Neuroticism, *E* Extraversion, *O* Openness to experience, *A* Agreeableness, *C* Conscientiousness

### Correlation analysis

**Fig. 1:**
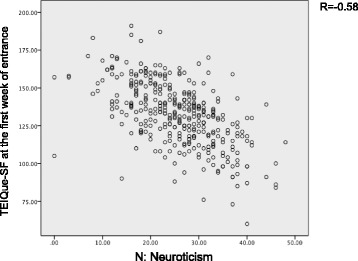
Pearson correlations between N and TEIQue-SF of all students at the first week of the university entrance resulted negative strong correlation (*R* = 0.583)

There was a weak correlation between TEIQue-SF and JSPE (*r* = 0.214, *p* < 0.01). Correlations between TEIQue-SF, JSPE and NEO-FFI and gender are shown in Table 4. TEIQue-S is strongly positively associated with E (*r* = 0.52, *p* < 0.01) and C (*r* = 0.42, *p* < 0.01) and moderately associate with A (*r* = 0.36, *p* < 0.01) and O (*r* = 0.24, *p* < 0.01). A strong negative correlation was found between TEIQue-SF and N (*r* = 0.583, *p* < 0.01 Fig. 1). Only weak positive correlations were found between JSPE and A (*r* = 0.26, *p* < 0.01), E (*r* = 0.25, *p* < 0.01) and O (*r* = 0.16, *p* < 0.05). Regarding effects of gender, two things were revealed. Firstly, N showed the strongest correlation of TEIQue-SF, followed by E, C and A in a similar pattern for both genders. Secondly, JSPE in males revealed correlated with A, E, O and N, but in females there was no correlation with NEO-FFI.

**Table 4:** Pearson Correlation of TEIQue-SF and JSPE with the NEO-FFI

TEIQue-SF	N	E	O	A	C
All (*n* = 357)	−0.58**	0.52**	0.24**	0.36**	0.42**
Male (*n* = 265)	−0.61**	0.58**	0.29**	0.39**	0.46**
Female (*n* = 92)	- 0.59**	0.46**	0.18	0.44**	0.40**
Empathy	N	E	O	A	C
All (*n* = 357)	−0.09	0.25**	0.16^*^	0.26**	0.06
Male (*n* = 265)	- 0.17**	0.32**	0.21^*^	0.33**	0.09
Female (*n* = 92)	0.14	0.02	0.02	0.14	−0.06

*TEIQue-SF* Trait Emotional Intelligence Questionnaire-Short Form, *JSPE* Jefferson Scale of Physician Empathy-student version, *N* Neuroticism, *E* Extraversion, *O* Openness to experience, *A* Agreeableness, *C* Conscientiousness

**p < 0.01 *p < 0.05

### Comparison analysis

Our analysis revealed no gender differences and similarities in TEIQue-SF, whereas there were significant but small effect size in gender difference for JSPE and 2 of 5 personality factors (A and N) with females recording higher scores (Table 5).

**Table 5:** Mean scores of TEIQue-SF, JSPE and NEO-FFI in gender and gender comparison and Cronbach α

*n* = 357	α value in this study	α value in original English	Male (SD) *n* = 265	Female (SD) *n* = 92	t value	df	*p* value	Effect size^a^
TEIQue SF	0.86	0.88	133.40 (20.72)	131.13 (21.15)	0.89	355	ns	0.11
JSPE	0.87	0.80	83.19 (9.39)	85.91 (7.22)	−2.87	355	0.01	0.30
N	0.85	0.83	25.23 (8.13)	27.33 (7.32)	−2.29	355	0.02	0.27
E	0.84	0.78	27.15 (7.07)	27.15 (7.07)	−0.79	355	ns	0.11
O	0.71	0.75	30.94 (5.79)	32.00 (6.06)	−1.46	355	ns	0.19
A	0.73	0.68	31.64 (5.36)	33.14 (5.43)	−2.29	355	0.02	0.28
C	0.81	0.77	29.03 (6.61)	29.97 (6.73)	−1.16	355	ns	0.15

*TEIQue-SF* Trait Emotional Intelligence Questionnaire-Short Form, *JSPE* Jefferson Scale of Physician Empathy-student version, *N* Neuroticism, *E* Extraversion, *O* Openness to experience, *A* Agreeableness and *C* Conscientiousness, *SD* Standard Deviation, *ns* not significant, *df* degree of freedom

^a^Cohen’s d

Cronbach alpha (Table 5) showed reliability both with the Japanese versions and with original English version. All alpha values in our study were found to be either good or acceptable (α range from 0.71 to 0.87), and were as high or higher than original English versions [13, 20, 40]. Thus, there was high confidence that all 3 Japanese versions of the questionnaires were reliable.

### Hierarchical multiple regression analysis

Hierarchical multiple regressions analysis showed that personality measures, except A, were significant predictors of TEIQue-SF (Table 6). N had the largest independent negative contribution (β = − 0,38), while E, C, O and JSPE had small positive contributions (β = 0.32, 0.25, 0.15 and 0.15, respectively). As for JSPE, smaller contributions were found for A, TEIQue-SF and N, but the other variables showed no contributions. However, gender difference contributes neither to EI nor to empathy. Effect size of the regression analysis were large for both.

**Table 6:** Results of hierarchical multiple Regression of TEIQue-SF and JSPE on variables and effect size

Dependent variable									
TEIQue-SF					JSPE				
Independent variable	β	*t value*	*p value*	Effect size^a^	Independent variable	β	*t value*	*p value*	Effect size^a^
Gender	−0.06	−1.72	0.09	0.60	Gender	0.09	1.79	0.07	0.22
N	−0.38	−10.52	0.00		N	0.17	3.04	0.01	
E	0.32	7.89	0.00		E	0.07	1.21	0.23	
O	0.15	3.89	0.00		O	0.09	1.61	0.11	
A	0.04	0.84	0.40		A	0.24	4.10	0.00	
C	0.25	6.68	0.00		C	−0.10	−1.75	0.08	
JSPE	0.15	3.85	0.00		TEIQue-SF	0.28	3.85	0.00	
Model	R	R^2^	Adjusted R^2^	*F* (df)	Model	R	R^2^	Adjusted R^2^	*F* (df)
	0.77	0.60	0.59	73.76 (7350)	0.47	0.22	0.21	14.24 (7350)	

*TEIQue-SF* Trait Emotional Intelligence Questionnaire Short Form, *JSPE* Jefferson Scale Physician Empathy student version, *N* Neuroticism, *E* Extraversion, *O* Openness, *A* Agreeableness, *C* Conscientiousness, *df* degree of freedom, *ns* not significant

^a^Cohen’s d

## Discussion

Empathic communication is important for patient-physician relationships. However previous studies report that empathy declined during the course of academic medical school training [41–44]. Recently, EI and empathy in medical students and physicians have been investigated to find out what factors, such as personality, gender, experience, influence on their empathic perceptions [26, 30, 42, 45, 46]. This study explored the association between EI, empathy and personality to find out what is the biggest factor for Japanese medical students’ empathic perceptions at a very early period of their medical school training, and to capture their characteristics to aid in developing a new communication program.

### Association between EI and empathy

This study is the first investigation of associations between EI, empathy and personality in Japanese medical students. Our study indicates that there is a weak association between EI and empathy. However, the results of regression analysis imply EI and empathy contribute to increase empathic ability. Mixed results have been reported for the correlation between EI and empathy [30, 43, 47]. One reason for these conflicting results might be due to differences in testing methods and measures [43]. JPSE measures predominantly cognitive empathy and some would argue that there is a strong implied normative aspect to JSPE in that it may indicated how doctors “should” behave whereas TEIQue-SF is measured in terms of “I can do” confidence to succeed in specific emotional situations or accomplish a task. Thus, while medical students at this stage may understand the value of empathic behavior, they may lack the confidence or resources to implement it. It may be that stronger associations would have been observed if a different measure of empathy had been used [47].

### Association between EI and personality

The most interesting finding in our study was the strong negative correlation found between EI and N (Fig. 1). In addition, medical students with higher EI showed higher correlation with E and C. A recent study of EI among medical students in the USA, showed strong positive association with E, moderate positive association with C, O and A and weak negative association with N [30]. Comparing this study to ours, it may be concluded that there are cultural differences between USA and Japan for the association between personality and EI. Hofstede G et al. indicated that culture and personality are not dependent, because to some extent personality is influenced by national culture [48]. Further studies are needed to compare associations between personality and EI in medical students in other counties.

There are two possible reasons to explain a higher association between N, E, C and EI, one is the entrance examination system and the other is students’ narrow view of the world. In Japan, the pathway to being accepted into medical schools begins early in childhood, with students devoting themselves to many hours of study in junior or senior high school, or even in elementary school, in order to pass the rigorous entrance exams. For many, this means restricted time to interact with their peers. The entrance examinations for Japanese medical schools strongly emphasis science and math, rather than the humanities or arts [24, 29], and voluntary activities are not valued, in contrast to medical school applicants in the USA or other Western countries. Thus, while Japanese medical students at orientation camp are often confident, social and energetic, leading to EI being highly associated with E and C, they tend to have a narrow view of the world, having focused on intense studying, with social interactions limited to family and peer interactions more through social media rather than face to face [49, 50]. A consequence of this is that Japanese medical students tend to have great anxiety about developing new peer relationships, and interacting with patients or other strangers. Thus, they have relatively high N scores, and tend to have decreased EI. The regression analysis shows that N has the strongest impact on EI, negatively affecting it. Thus, reducing N may be the key to increase EI.

The evidence suggests that O and C are more predictive for medical students’ competence in clinical tasks [51], A and O are more favourable for building better patient-physician relationships [52] and training proficiency [53], and C is important for task completion [54]. However, due to academic demands that reduce peer group interactions, Japanese medical students are less able to accurately judge and react to other people’s emotions. [49]. These may result in medical students’ A and O scores being weakly associated with EI. This emotional deficiency may be improved by increasing students’ face to face interaction with diverse people [50], and improve A and O scores during 6 years of their medical school training.

### Association between empathy and personality

In contrast to EI, there are no associations between empathy and N, C and weak associations between empathy and E, A. Contrasting to our observations, there is evidence of a greater association between empathy and personality than between EI and personality [22, 30, 33]. Though each study used the same JSPE scale, Japanese medical students’ empathy associated weakly with E and A. It may be because cognitive empathy arises from understanding [35] of how physician should behave [47], so while Japanese medical students understand the importance of empathy very well, they may be less adept at putting this knowledge in to practice. Although it requires further investigation, we hypothesize that cognitive emotional understanding (JSPE) is less influenced by personality than by a “can do” aspect of emotional self-efficacy (TEIQue-SF).

### Gender differences

Our study revealed significant personality differences by gender, with females being more N and A, although the effect size for both are relatively small. Consistent with our findings, a meta-analysis study showed that females had higher anxiety and agreeability scores [55]. Females tend to express emotion and are more skillful with interpersonal communication than males [56]. This is especially true for Japanese females, who tend to be more sensitive to interpersonal relationships in a cooperative group, and tend to compare themselves more with other group members [57]. Thus, female Japanese medical students possess higher empathy and more agreeability scores than males.

Our results showed no significant gender differences for EI. Other studies of medical students have reported conflicting findings in respect of EI and gender [19, 44, 58, 59]. When controlling for gender and personality in the regression analysis, undertaken in this study, gender did not affect EI and empathy, rather personality was the most important factor. This result implies that personality influences students’ empathic perceptions.

### Cultural differences

JSPE scores in our results showed that empathy scores for female students were significantly higher than males, consistent with previous studies [28–30, 43, 58, 60, 61]. In addition, JSPE scores of medical students in Western countries were higher than those of medical students in Asian countries [24, 28–30, 62]. We used the JSPE with 5-point Likert Scale, as used in a study of Scottish medical students that showed that for 1st year medical students, female students’ empathy score was significantly higher than males [58], consistent with our results. In a Japanese cross-sectional study [44], medical students’ JSPE scores declined by the 4th academic year. It is likely that the timing of our survey of our study population, i.e. students at the very beginning of their medical school training, might affect these scores. As Kataoka notes, medical students mostly study basic science in the 1st and 2nd medical year [29] thus, it is possible that focusing on these science-oriented curricula may decrease their empathy.

We investigated only Japanese students, with none from other cultures, so we cannot really comment on cultural differences. However, it is important to note that several factors may give rise to different scores when comparing studies from different countries, for example there may differences in research designs, in timing of when studies were conducted, or differences in medical school curricula.

It is thought that Asian medical students’ EI is lower than Western ones. Trait Emotional Intelligence represents “trait emotional self-efficacy”. Because Bandura explains that self-efficacy consists of 4 information sources, with fulfillment experience, surrogate experience, linguistic persuasion, and physical/emotional reaction, self-efficacy influencing trait EI [63]. A study comparing self-efficacy between individuals in different countries show that Japan was the lowest among 25 nations [64], with 18-year-old Japanese scoring 20.23 out of 40 compared to 31.13 for Dutch and 30.19 for British. A study of Trait EI showed a similar tendency, with EI scores for Japanese individual (132.8) being much lower than Dutch (160) [26] and Scottish (157) [65]; our results are consistent with these studies. There are reasons for these cultural differences, for example, Scholz et al. indicated that with strong collectivist culture that exist in Asian societies like those of Japan or Hong Kong, values are placed on efforts and diligence rather than individual’s abilities, resulting in a lower emphasis on individualism, and thereby producing a lower self-efficacy score [64]. Also, Markus et al. concluded that while Americans have mutually independent self-views, Asians have a mutual collaborative self-view, so emphasizing harmony with others, they tend to have self-critical attitudes [66]. These cultural concepts may result in lowered EI.

### Limitations

The data collect in this study was from different cohorts of 1st year students (over 4 years) at only one university. A gender imbalance in our study population may raise some validity and generalizability concerns. Future studies should include more female students in the cohort. An addition limitation was that data was collected at one time point in the medical students’ academic career; future studies should follow individuals over the course of the medical school training. Further investment in training programs designed to manage emotion might be helpful in reducing medical students’ stress [67]. It is important to assess how communication training could enhance medical students’ EI and empathy, thereby reducing N and increasing A, O and C scores, to achieve the desired objective of producing more empathic physicians.

## Conclusion

Our study investigated the associations between EI, empathy and personality in Japanese 1st year medical students at orientation camp. Females had significantly higher in N, A and empathy scores than males. We observed weak associations of empathy and EI. Medical students’ EI was strongly negatively associated with N and positively associated with E and C. However, when controlling for gender and personality in the regression analysis, gender did not influence EI and empathy, rather personality was the most important factor. The results imply that N is a major factor that negatively affects EI for Japanese 1st year medical students. Approaches designed to enhance EI might be helpful in reducing N and increasing A and O. Medical students’ EI may be enhance with thoughtful training, taking into account students’ personalities.

## Abbreviations

A: Agreeableness
AAMC: Association of American Medical Colleges
ACGME: Accreditation Council for Graduate Medical Education
C: Conscientiousness
E: Extraversion
EI: Emotional Intelligence
JSPE: Jefferson Scale of Physician Empathy- student version
N: Neuroticism
NEO-FFI: NEO-Five-Factor Inventory
O: Openness to experience
TEIQue-SF: Trait Emotional Intelligence Questionnaire-Short Form
